# The Effect of Impaired Cerebral Autoregulation on Postoperative Delirium in Neonates and Infants After Corrective Cardiac Surgery: A Study on Modifiable Risk Factors for Delirium

**DOI:** 10.31083/RCM37292

**Published:** 2025-06-30

**Authors:** Yordan H. Georgiev, Marcel Methner, Maximilian Iller, Juliane Engel, Jörg Michel, Johannes Nordmeyer, Felix Neunhoeffer

**Affiliations:** ^1^Department of Pediatric Cardiology, Pulmonology and Pediatric Intensive Care Medicine, University Children's Hospital Tübingen, 72076 Tübingen, Germany

**Keywords:** cerebral autoregulation, delirium, congenital heart disease, corrective cardiac surgery, modifiable factors

## Abstract

**Background:**

The risk factors for developing postoperative pediatric delirium (PD) are multifactorial and include underlying conditions, cyanosis, surgery, intensive care stay, analgesia used for sedation, and withdrawal symptoms. Disturbed cerebral autoregulation in children with congenital heart disease (CHD) can lead to hyper- and hypoperfusion states of the central nervous system and is potentially associated with poor neurological outcomes. Our study aimed to investigate whether disturbed cerebral autoregulation postoperatively is associated with the onset of PD in children with CHD.

**Methods:**

We conducted a prospective observational study in neonates and infants undergoing corrective surgery for CHD via cardiopulmonary bypass (CPB). Cerebral regional oxygen saturation (rSO2) and mean arterial pressure (MAP) were measured within the first 24 hours after surgery in the pediatric intensive care unit (PICU). The cerebral oximetry index (COx) was calculated from these parameters using ICM+ software. A COx ≥0.4 was considered indicative of impaired autoregulation. Delirium symptoms were assessed using the Sophia Observation of Withdrawal–Pediatric Delirium (SOS-PD) score.

**Results:**

Cerebral autoregulation was evaluated postoperatively at the bedside of 49 neonates and infants (22 males, 44.9%, vs. 27 females, 55.1%) between January 2019 and April 2023. The median age of the patients was 134 days (interquartile range (IQR): 49.5–184 days), the median weight was 5.1 kg (IQR: 4.0–6.3 kg), and the monitoring duration was 23.0 hours (IQR: 20–24.5 hours). In total, 27/49 (55%) patients developed postoperative PD during their stay in the PICU. There was no statistically significant difference in the duration of globally impaired autoregulation between the delirious and non-delirious groups (14.5% vs. 13.9%, *p* = 0.416). No evidence was found supporting the effect of MAP outside the lower and upper limits of autoregulation for the onset of postoperative delirium (*p* = 0.145 and *p* = 0.904, respectively). Prolonged mechanical ventilation, longer PICU stay, and higher use of opioids and benzodiazepines were observed in the delirious group.

**Conclusion:**

Our findings suggest that impairment of cerebral autoregulation cannot solely explain the higher rate of PD in children undergoing congenital cardiac surgery. Rigorous hemodynamic management may potentially minimize the impact of cerebral hypo- or hyperperfusion states during the postoperative period, preventing their harmful effects. Additional studies with a larger sample size are needed to confirm the hypothesis and current findings.

## 1. Introduction

Pediatric delirium (PD) is a serious condition characterized by the sudden onset of cognitive impairment caused by an underlying medical condition. It can lead to increased morbidity and mortality [1].

The incidence of PD in pediatric intensive care units (PICUs) varies between 17–26% [2, 3, 4]. In children who underwent cardiac surgery on cardiopulmonary bypass (CPB), the incidence is higher, reaching levels of 40–67% [5, 6, 7].

Various scoring systems have been developed over the last 10 years to measure PD - the Sophia Observation Withdrawal Symptoms scale Pediatric Delirium scale (SOS-PD scale) [8], and the Cornell Assessment of Pediatric Delirium [9], focusing on the early diagnosis of PD. Unfortunately, despite improved screening methods, this issue is underestimated in the pediatric population [10, 11]. The SOS-PD scale demonstrates high sensitivity and specificity, with values of 92.3% and 96.5%, respectively, when compared to the clinical diagnosis made by psychiatrists. In addition, this scale can be easily integrated into clinical practice and administered by nurses [8].

Prevention of PD remains a primary and important challenge. The implementation of a delirium bundle in the PICU, including pharmacological and non-pharmacological procedures, has demonstrated efficacy in reducing the rate of delirium in children who underwent congenital heart surgery [12]. It is a crucial component of the comprehensive concept, known as the ABCDEF bundle. It includes preventative strategies that aim to decrease the incidence of post-intensive care syndrome (PICS) for both patients and families [13].

Various factors in this patient population contribute to the onset of delirium –age less than 2 years, cyanotic congenital heart disease (CHD), prolonged mechanical ventilation, use of benzodiazepines and opioids, and surgical complexity [5, 6, 7]. However, there is still a knowledge gap, as not all the factors have been sufficiently investigated. In special populations, such as children with CHD, where the incidence of PD is much higher, other factors need to be explored. Studies in adult patients undergoing cardiac surgery demonstrated that impaired cerebral autoregulation in the operating room and intensive care unit may play a crucial role in the development of postoperative delirium [14, 15], and can play an important role in the pathophysiology of postoperative delirium in adult patients.

The implementation of near infrared spectrometry (NIRS) provides continuous, non-invasive neurophysiological monitoring. This enables determination of cerebral oximetry index (COx) and hemoglobin volume index HVx, which indirectly correlate with changes in cerebral blood flow (CBF) and cerebral blood volume (CBV), respectively [16]. These surrogate parameters contribute to identifying changes in cerebral autoregulation and assessing of its integrity, without the need for invasive procedures (aside from invasive blood pressure measurement) [17, 18]. COx is one of the most commonly used surrogate parameters for cerebral autoregulation in clinical practice. In cases of intact autoregulation, there is minimal correlation between cerebral regional oxygen saturation (rSO2) and mean arterial pressure (MAP), while impaired autoregulation results in a stronger correlation, indicating a higher index. However, there is still no consensus regarding the COx threshold at which autoregulation is considered impaired [17, 19, 20].

Since PD remains a significant issue in children with CHD, it is crucial to identify additional modifiable risk factors that are related to PD. There is a lack of studies investigating cerebral autoregulation in the PICU as a risk factor for PD. We aimed to investigate whether excessive blood flow or impaired autoregulation during the first 24 hours after corrective cardiac surgery plays a role in the development of PD. We hypothesized that children with impaired autoregulation and/or prolonged periods of excessive cerebral blood flow in the first 24 hours postoperatively are at an increased risk of developing PD.

## 2. Materials and Methods

### 2.1 Study Design and Setting

This single-center prospective, observational study was performed at the 14-bed tertiary PICU of the University Children’s Hospital, Tübingen, Germany. The study included patients with CHD under 1 year of age who were admitted to the PICU from January 2019 until April 2023 after undergoing corrective cardiac surgery on CPB. There were three measurement periods: February 2019 to January 2020, May 2021 to January 2022, and November 2022 to April 2023.

### 2.2 Data Collection and Definitions

All patients received treatment according to a standardized protocol. Postoperative hemodynamic therapy was managed with norepinephrine, milrinone, and/or adrenaline. The hemodynamic status was actively monitored using invasive arterial blood pressure, central venous oxygen saturation, serum lactate, and diuresis parameters, while perfusion status was assessed by monitoring capillary refill time. Therapeutic goals were MAP >45 mmHg in children <6 months and MAP >50 mmHg in children >6 months, a difference between arterial oxygen saturation and central venous saturation of 30%, diuresis >2 mL/kg/h, capillary refill time <2 s, and lactate <2 mmol/L [21]. In all patients, a major intracerebral hemorrhage was ruled out postoperatively using transfontanelle ultrasound.

The vasoactive inotropic score (VIS) was calculated using the following formula: 100 × epinephrine dose [µg kg^-1^ min^-1^] + 50 × Levosimendan dose [µg kg^-1^ min^-1^] + 10 × Milrinone dose [µg kg^-1^ min^-1^] + 100 × Norepinephrine dose [µg kg^-1^ min^-1^]). Measurements were recorded at the beginning and end of the measurement period and are reported as average values.

The transfusion trigger for patients after corrective cardiac surgery at our institution is hemoglobin (Hgb) <8.0 g/dL.

All children were intubated on admission to the PICU. Patients were extubated after a period of cardiopulmonary and respiratory stability, and adequate preparation using an extubation checklist.

#### 2.2.1 Parameters of Cerebral Autoregulation

For NIRS measurements, the two-channel INVOS™ 5100C Cerebral/Somatic Oximeter (Medtronic, Inc., Minneapolis, MN, USA) with OxyAlert™ NIRSensor was utilized, placed on the forehead, lateral to the midline. This enabled an assessment of rSO2 using a two-wavelength LED source (730 and 810 nm) and two photodiode detectors with source-detector separations of 30 and 40 mm [17]. This allowed the optical absorption coefficient differences between reduced and oxygenated hemoglobin and the local concentrations of these two types of hemoglobin to be determined [22]. RSO2 is inversely proportional to the transmittance of light with a wavelength of 805–810 nm, which is isosbestic to both reduced and oxygenated hemoglobin [21].

The measurement of CBF involved a multi-step process and was based on invasive MAP. ICM + software (Cambridge Enterprises, Cambridge, UK) was employed to ensure alignment between the rSO2 and MAP. Monitoring parameters were digitally sampled at 100 Hz [17]. COx was calculated with a continuous moving Pearson correlation between rSO2 and MAP. Ten-seconds averaged values from paired sets, each lasting five minutes, were utilized for calculating the COx. Thus, COx represents continuous variables within a range of –1 to +1. The assumption underlying COx as a surrogate parameter of CBF is that fluctuations in CBF, induced by autoregulatory vasoconstriction and vasodilatation, are proportionate to changes in rSO2 [21]. However, the relationship may not always be linear, especially in pathological conditions where autoregulation is impaired. In such cases, COx values may fail to accurately reflect changes in CBF, which could make them less reliable in certain situations.

The results were graphically represented using a U-curve to depict the MAP at various COx levels [23]. This was done at the end of the measurement period after removing artifacts, such as those caused by arterial blood gas sampling. The earliest available comprehensive graphic was selected, beginning from the earliest time point that allowed for a valid analysis. A time window of 8 hours was applied to visualize the U-curve. Below and above the optimal mean arterial pressure (MAPopt), the U-curve rises towards the COx cutoff (0.4), which defines the lower limit of autoregulation (LLA) and the upper limit of autoregulation (ULA) [17].

The COx values were categorized into bins based on MAP, with each bin representing a 5-mmHg interval. Bar graphics were created for each bin. MAPopt was identified as the bin with the most negative index (nadir). The lower limit of autoregulation was determined as the bin where COx ≥0.4 and the bar graph showed increasing index values with decreasing MAP. The upper limit of autoregulation was defined as the bin where COx ≥0.4 and the bar graph showed increasing index values with increasing MAP [17, 21].

Since CBF is relatively constant between the LLA and ULA, it can be assumed that cerebral autoregulation remains intact within this range [24]. Therefore, we calculated the period of time in which the patient experienced an impaired autoregulation in relation to the total duration of cerebral autoregulation monitoring as a percentage.

#### 2.2.2 Parameters of Sedation and PD

All patients were managed according to our institutional standardized, goal-directed, nurse-driven analgesia and sedation protocol, which has been previously described in detail [25].

Delirium symptoms were assessed at least every 8 h by the responsible nurse using SOS-PD. PD was diagnosed by a cut-off score ≥4 [8, 26]. The score was obtained at least once a shift or more frequently in case of a change in the patient´s condition. Based on these parameters, we were able to calculate the duration of delirium for every patient and as a percentage - the proportion of the time with symptoms of delirium in relation to the total time spent in the intensive care unit. We subcategorized the form of delirium into hypoactive, hyperactive and mixed form according to the criteria described in Diagnostic and Statistical Manual of Mental Disorders (DSM-5) [27].

### 2.3 Statistical Analisys

Statistical analysis was performed using the SigmaPlot, Version 13 (Systat Software, Inc., San Jose, CA, USA). Initially, a Shapiro-Wilk test was conducted to examine normal distribution. In cases where data exhibited normal distribution, mean and standard deviation (SD) were presented. Conversely, if a normal distribution was not observed, median and interquartile range (IQR) were reported. Categorical data are presented as frequencies and percentages. All *p*-values < 0.05 were considered statistically significant. Comparisons of 2 or more groups were performed using one way analysis of variance (ANOVA). For categorical variables Fisher’s exact test was used to analyze the differences between groups. A multivariable logistic regression analysis was performed to identify risk factors associated with impaired cerebral autoregulation.

## 3. Results

### 3.1 Recruitment of the Patients

**Fig. 1.**
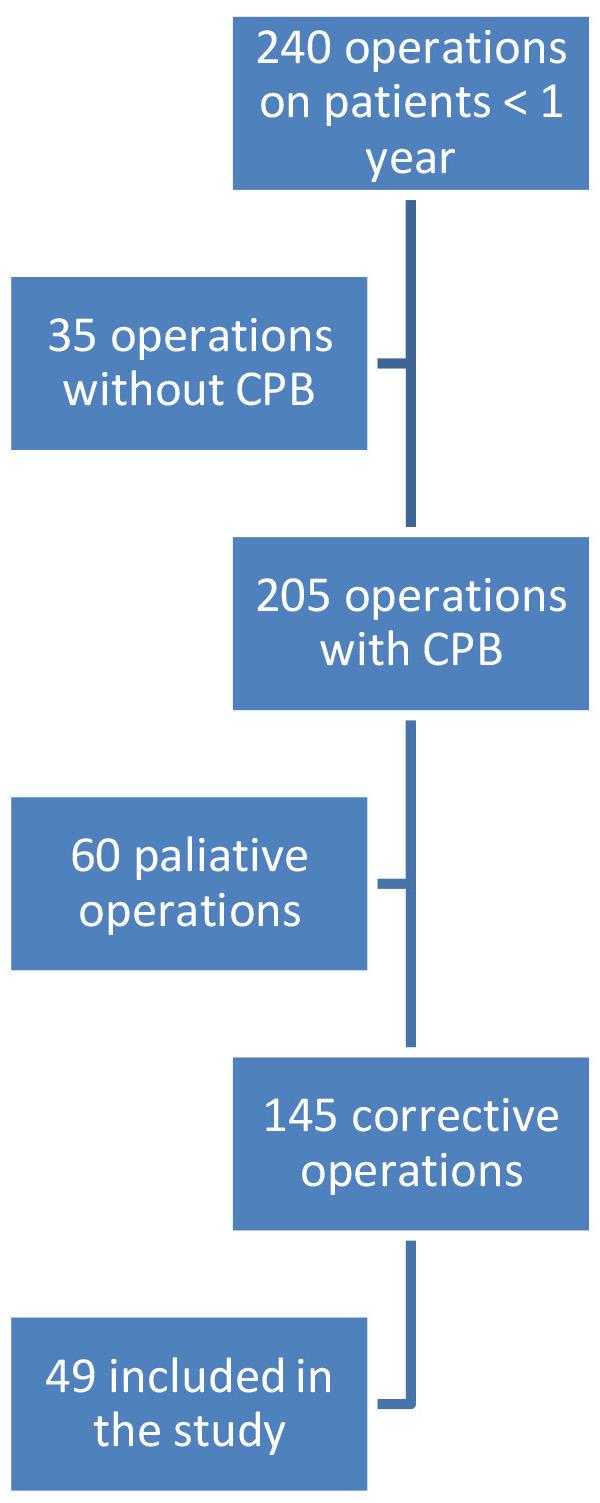
**Flow chart of the patients included in the study**. CPB, cardiopulmonary bypass.

During the study period, 240 operations were performed on children under 1 year of age, of which 205 were with the use of CPB. 145 were corrective surgical procedures. Due to technical reasons, measurements could not be performed on two consecutive days. Additionally, measurements could only be conducted when at least one of both examiners (MI and MM) was present in the clinic. Finally, 49 patients were included in the study (Fig. 1). We hypothesized that a total of approximately 50 patients would be sufficient to provide initial estimates of effect size and variability. This sample size allows for the detection of a moderate to large effect (Cohen’s h ≈ 0.57) with 80% power at a two-sided alpha of 0.05. While the study is not powered to detect small differences, it is intended to generate preliminary data to determine the design of future larger investigations.

### 3.2 Patient Characteristics

During the study period 49 measurements with a median duration of 23.0 (20–24.5) hours were performed in the PICU. The median age was 134 (49.5–184) days and the median weight was 5.1 (4.0–6.3) kg. All patients underwent corrective cardiac surgery and had following diagnoses: fourteen cases of Tetralogy of Fallot, eleven cases of ventricular septal defect, nine cases of atrioventricular septal defect, eight cases of transposition of the great arteries, five cases of aortic arch surgery, and two cases of truncus arteriosus communis. The data were divided into two subgroups based on whether the children experienced a postoperative delirium or not. The subgroup analysis is represented in Table 1.

**Table 1.** **Patent characteristics and subgroup distribution**.

Parameter	Total population (n = 49)	No delirium (n = 22)	Delirium (n = 27)	*p* value
Age [d] Median (IQR)	134 (49.5–184)	110.5 (12.8–162.8)	145 (102–188)	0.159
Male gender n (%)	22 (44.9)	10 (45.5)	12 (44.4)	1.000
Weight [kg] Median (IQR)	5.1 (4.0–6.3)	5.3 (3.5–7.8)	5.1 (4.0–5.6)	0.615
Measurement duration [h] Median (IQR)	23.0 (20–24.5)	23.0 (21.8–24.4)	23.0 (18–25)	0.809
Interval between operation conclusion and measurement onset [h]	4 (3–5.5)	3.3 (3–5)	4.5 (3.5–6)	0.079
CPB duration [min] Median (IQR)	111 (80–148.5)	118.5 (87.5–148.3)	105 (79–152)	0.851
Aortic cross-clamp time [min] Median (IQR)	90 (63.5–114)	92.5 (55.3–121.8)	90 (68–100)	0.880
Duration of PICU stay [d]	6 (4–10)	4 (3–7)	9 (5–14)	<0.001*
Duration of mechanical ventilation [h]	69 (24–143)	26 (16.3–99.1)	88 (48–166)	0.006*
Cumulative morphine [mg/kg]	3.7 (2.1–7.5)	1.8 (1.1–4.9)	5.2 (3.1–11.7)	<0.001*
Cumulative clonidine [mg/kg]	0.1 (0.05–0.3)	0.1 (0–0.2)	0.2 (0.1–0.4)	0.004
Cumulative midazolam [mg/kg]	0 (0–7.2)	0 (0–2.5)	4.2 (0–15.3)	0.023*
VIS	7.4 (4.3–12)	5.9 (2.3–6.5)	7.5 (6.5–14.4)	0.04*
Average lactate during the measurement period [mmol/L]	1.2 (0.8–1.7)	1.3 (0.9–1.8)	1.1 (0.8–1.6)	0.305
Central venous oxygen saturation [%]	58.2 ± 8.9	61.9 ± 6.2	56.1 ± 9.6	0.057

Continuous data are represented as median and interquartile range, categorical data are represented as frequencies and percentages. d, day; h, hour; min, minute; IQR, interquartile range; PICU, pediatric intensive care unit; VIS, vasoactive inotropic score. * All *p*-values < 0.05 were considered statistically significant.

The two subgroups exhibited homogeneity in terms of age, gender, and weight, as there was no statistically significant difference observed between these parameters in either group. The measurement duration was similar for both groups, with a median of 23 hours, *p* = 0.809. There was no statistically significant difference in the duration of CPB or aortic cross-clamp times between both groups, with *p*-values of 0.851 and 0.880, respectively. Both groups exhibited a difference in the duration of PICU stay and mechanical ventilation, and they were significantly longer in the delirium group compared to the non-delirium group (9 vs. 4 days, *p* < 0.001 and 88 vs. 26 hours, *p* = 0.006, respectively).

Other known delirium-associated factors, such as cumulative doses of opioids and midazolam, also demonstrated statistically significant differences between delirious and non-delirious groups was 5.16 vs. 1.81 mg/kg, *p* < 0.001 and 4.17 vs. 0 mg/kg, *p* = 0.004 mg/kg, respectively.

### 3.3 Parameter of Cerebral Autoregulation

**Fig. 2.**
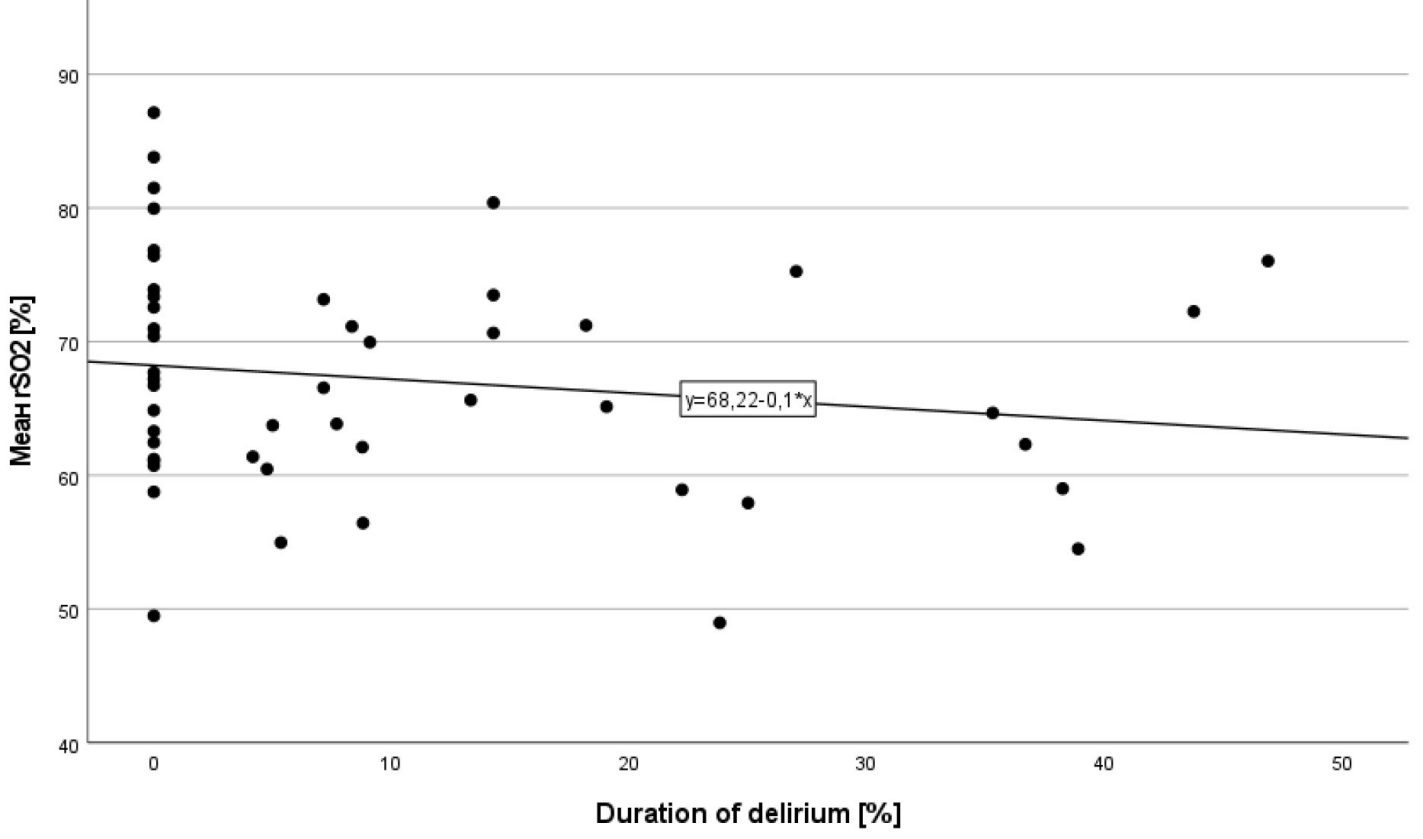
**Scatter plot of mean rSO2 for each patient in correlation with duration of delirium**.

Table 2 represents the parameter of cerebral autoregulation for the overall population in both subgroups. For every child, a mean rSO2 for the duration of the entire measurement could be calculated. The Shapiro-Wilk test showed a normal distribution (0.938), the mean rSO2 was 67.2 ± 8.6%. There was no statistically significant difference in this parameter between the non-delirious and delirious group (69.6 ± 9.2 vs. 65.2 ± 7.6%, *p* = 0.075). Fig. 2 demonstrates the mean rSO2 for each patient included in this study in correlation with duration of delirium.

**Table 2.** **Parameter of cerebral autoregulation for the entire population and both subgroups—without delirium and with delirium**.

Parameter	Total population (n = 49)	No delirium (n = 22)	Delirium (n = 27)	*p* value
rSO2 [%]	67.2 ± 8.6	69.6 ± 9.2	65.2 ± 7.6	0.075
Mean (SD)				
MAPopt [mmHg]	56.1 ± 6.5	57.1 ± 7.2	55.3 ± 5.8	0.336
Mean (SD)				
LLA [mmHg]	46.0 ± 4.7	46.5 ± 5.2	45.7 ± 4.4	0.600
Mean (SD)				
ULA [mmHg]	65.6 ± 7.9	66.7 ± 7.0	64.8 ± 8.7	0.531
Mean (SD)				

As there was a normal distribution, the data are represented as mean and standard deviation. MAPopt, optimal mean arterial pressure; LLA, lower limit of autoregulation; ULA, upper limit of autoregulation; rSO2, cerebral regional oxygen saturation; SD, standard deviation.

**Fig. 3.**
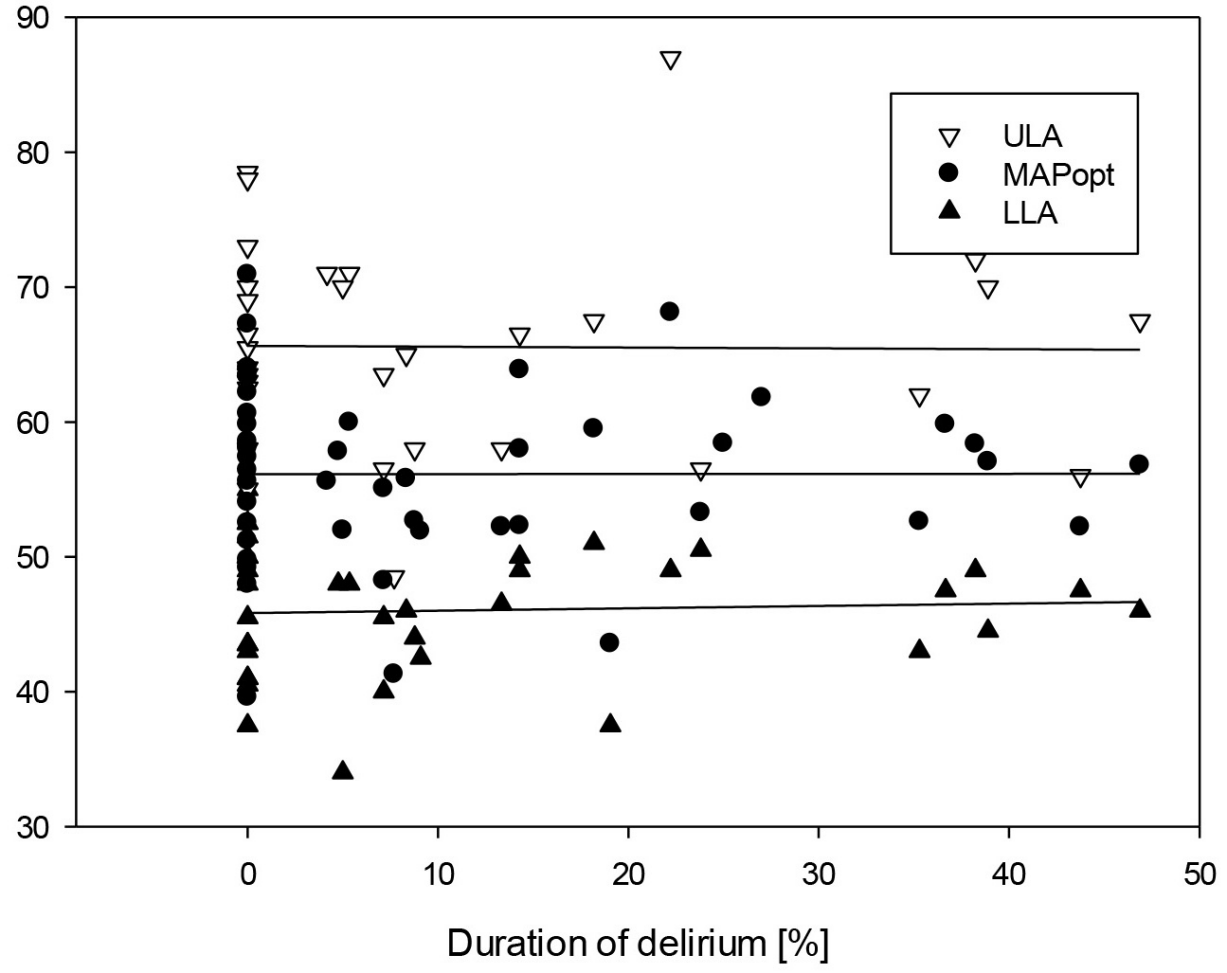
**Scatter plot of all measured ULA, LLA and MAPopt in correlation with the duration of delirium**.

MAPopt could be determined in 21/22 and 26/27 of the children in the non-delirious and delirious groups, respectively. It demonstrated similar results in both subgroups: 57.1 ± 7.2 vs. 55.3 ± 5.8, *p* = 0.336 (Fig. 3).

The LLA could be determined in 37 out of 49 cases—16 in the non-delirious group and 21 in the delirious group. There was no statistically significant difference between both groups (*p* = 0.6).

The ULA could be determined in 31 out of 49 patients—13 in the non-delirious group and 18 in the delirious group, without a statistically significant difference (*p* = 0.531).

In the multivariable logistic regression model including rSO2, ULA mean, LLA mean, and MAPopt, none of the variables reached statistical significance—ULA mean (OR 1.14, 95% CI: 0.67–1.92, *p * =  0.636), LLA Mean (OR 1.35, 95% CI: 0.76–2.38, *p * =  0.308), and MAPopt (OR 0.61, 95% CI: 0.21–1.74, *p*  =  0.354). However, there was a trend towards a protective effect of higher rSO2 values on the occurrence of PD, with an odds ratio of 0.89 (95% CI: 0.78–1, *p* = 0.062).

In all but 8 patients, a mixed form of PD with fluctuating phases between hyperactive and hypoactive delirium was observed. In the remaining 8 patients, only hyperactive delirium was noted. There were no statistically significant differences in autoregulation parameters between the two groups: LLA hyperactive 45.8 (41.9–48.3) mmHg vs. mixed 47.5 (44.0–49.0) mmHg, *p* = 0.435; ULA hyperactive 60 ± 7.5 mmHg vs. mixed 66.7 ± 8.6 mmHg, *p* = 0.15; MAPopt hyperactive 52.5 ± 5.6 mmHg vs. mixed 56.5 ± 5.5 mmHg, *p* = 0.104; rSO2 hyperactive 69.5 ± 6.1% vs. mixed 63.4 ± 7.6%, *p* = 0.053.

### 3.4 Subgroup Analysis of Globally Impaired Autoregulation 

**Fig. 4.**
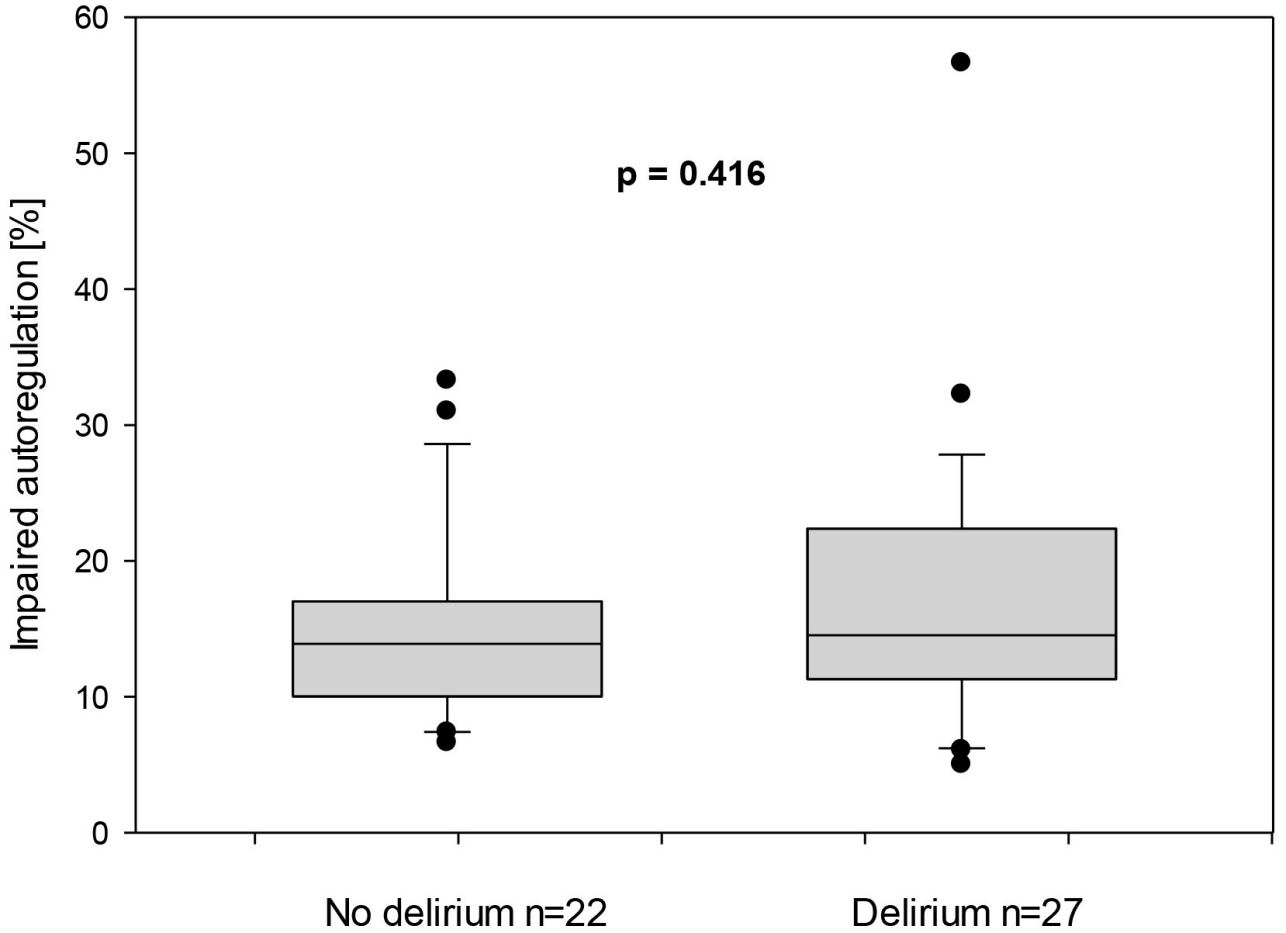
**Duration of impaired autoregulation represented in percentage in children with and without postoperative delirium**.

The duration of impaired autoregulation in relation to the total measurement duration was 14.5% for children with postoperative delirium, compared to 13.9% for those without (*p* = 0.416) (Fig. 4).

**Fig. 5.**
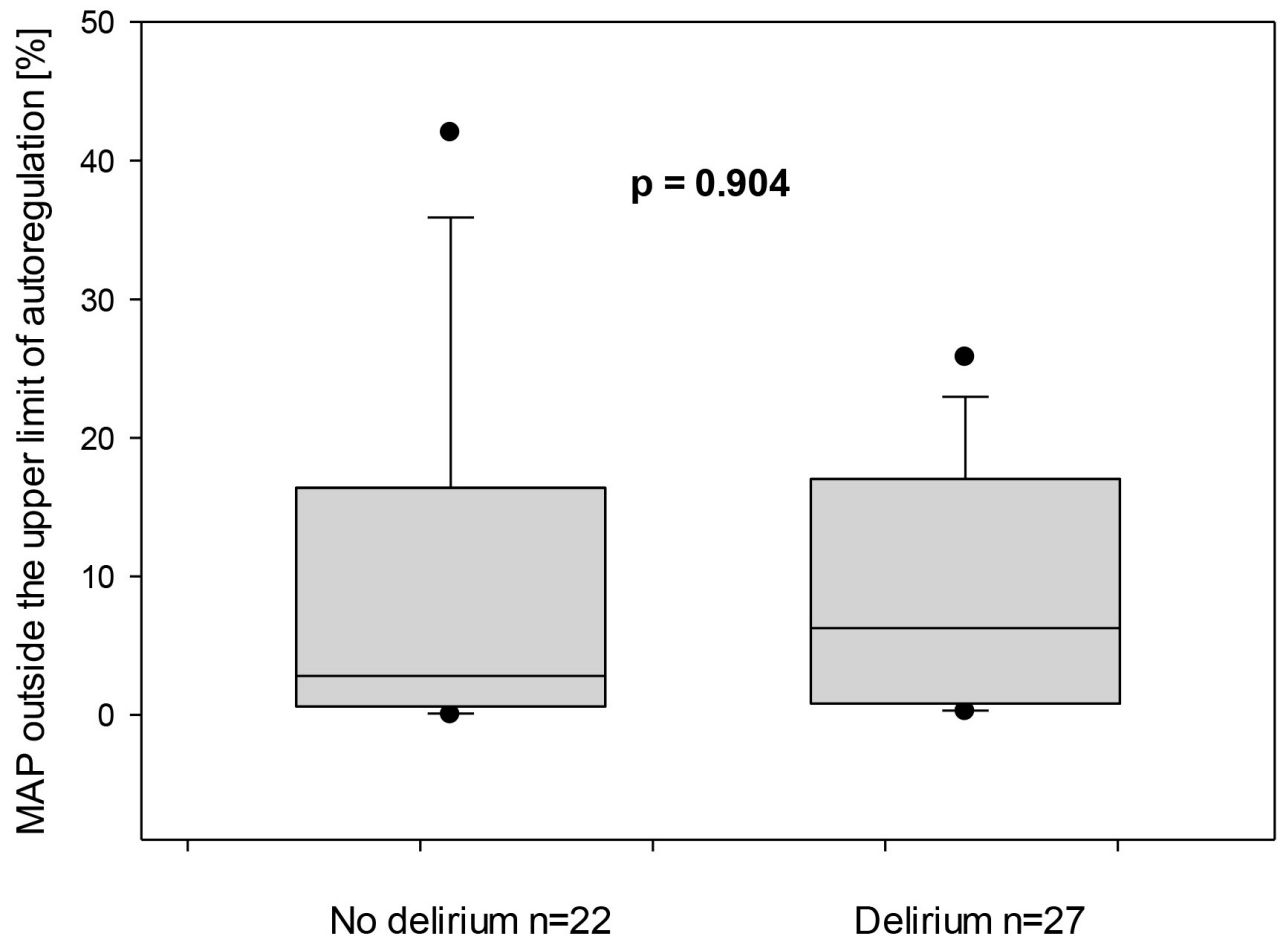
**Mean arterial pressure (MAP) outside the upper limit of autoregulation for both subgroups—with and without delirium**. On the X-axis, the proportion of time in which the MAP was outside the ULA is represented in relation to the entire monitored duration.

**Fig. 6.**
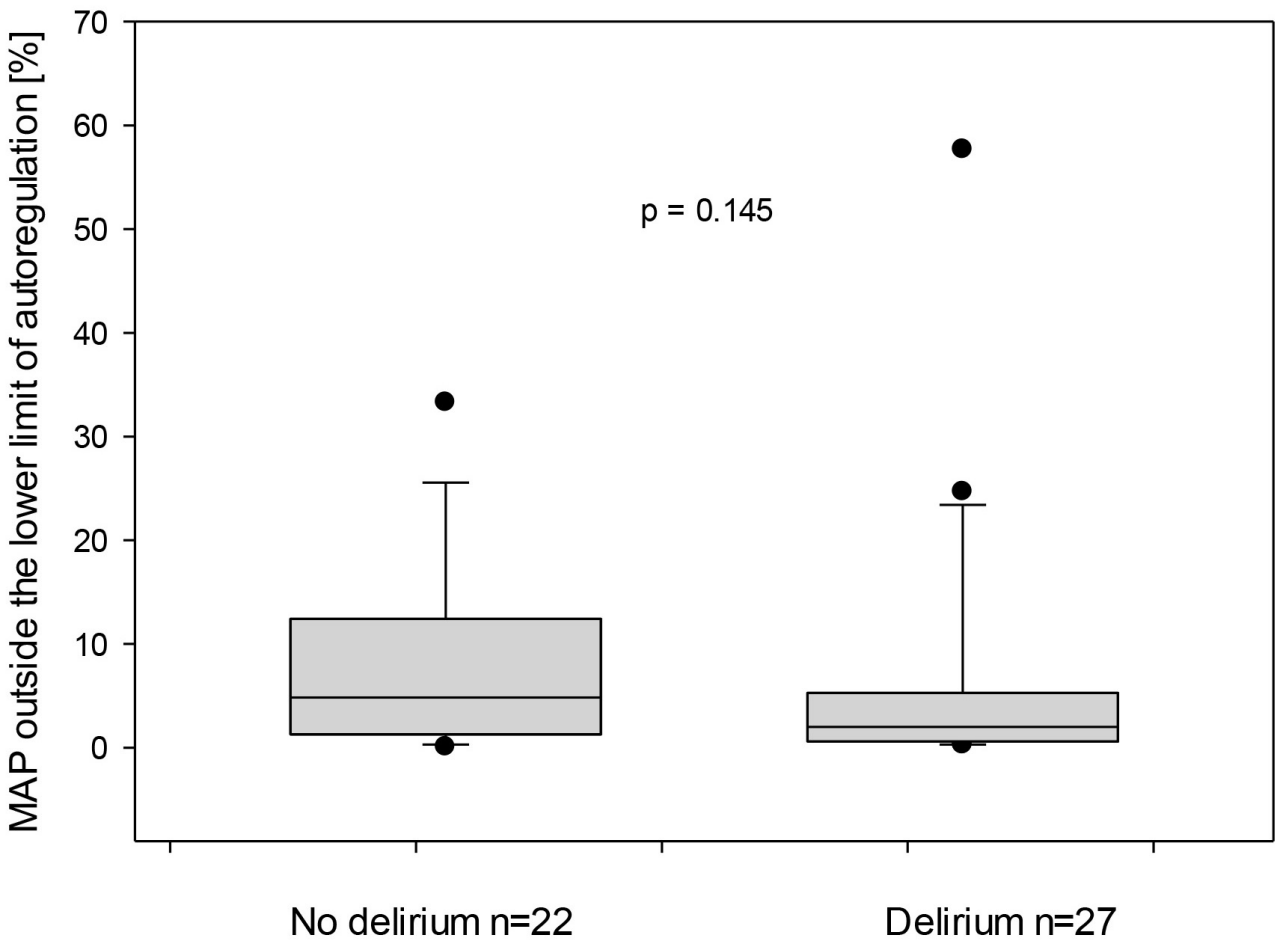
**Mean arterial pressure (MAP) outside the lower limit of autoregulation for both subgroups—with and without delirium**. On the X-axis, the proportion of time in which the MAP was outside the LLA is represented in relation to the entire monitored duration.

The total duration of mean arterial pressure outside the upper limit of autoregulation in relation to the total measurement duration was similar for both non-delirious and delirious groups was 6.3 (0.8–17) vs. 2.8 (0.6–16.4)%, *p* = 0.904, respectively, Fig. 5. Similarly, the total duration of MAP outside the lower limit of autoregulation in relation to the total measurement duration demonstrated no statistically significant difference for both groups: 4.8 (1.3–12.4) and 2 (0.6–5.3) %, respectively, Fig. 6.

## 4. Discussion

In this study, we sought to identify another modifiable risk factor for the onset of PD after congenital cardiac surgery. However, our data demonstrated no direct correlation between impaired autoregulation and the onset of delirium.

In recent years, several studies showed controversial results in this area [14, 15, 19, 28]. Nakano *et al*. [15] observed a higher rate of globally impaired autoregulation among 134 adult patients following cardiac surgery in the intensive care unit, which was associated with a statistically significant higher incidence of delirium (*p* = 0.04). Conversely, in our study, we could not find a statistically significant difference (*p* = 0.416) between both groups.

Nakano *et al*. [15] also found that the MAP outside the ULA in operating room was also associated with a higher incidence of PD (*p* = 0.005). However, these findings could not be replicated later in the intensive care unit (ICU) (*p* = 0.07). In a systematic review on adults undergoing CPB, this has been identified as an independent risk factor for the development of postoperative delirium [29]. This demonstrates that intraoperative cerebral overflow may have a crucial role in the onset of delirium. Furthermore, this may play a primary role, rather than impaired autoregulation in the early postoperative stage.

Another recent study [28] of 51 children who underwent congenital cardiac surgery also found no association between the duration of impaired autoregulation at the first 96 hours postoperatively and the onset of PD.

Consistent with these results, in our study, the duration of impaired autoregulation and MAP outside the ULA were not associated with a higher incidence of postoperative PD (*p* = 0.416 and *p* = 0.904, respectively). These discrepancies in the studies demonstrate the complexity of PD and suggest that the development of postoperative PD cannot be explained solely by impaired autoregulation in the PICU. Since cerebral autoregulation tends to recover in most cases after CPB, any impairment may not be detected in the intensive care unit [29]. However, it is possible that the injury had occurred during the vulnerable intraoperative phase and could not be further established during the monitored time in the PICU. In our study, measurements of cerebral autoregulation began at a median of 4 hours after the operation had been completed. The exclusion of the very early postoperative phase might have led to underestimating the possible fluctuations in CBF immediately after the operation or during CPB.

It remains unclear whether cerebral autoregulation in adults was already impaired in the preoperative period, as certain cardiac conditions can lead to low cardiac output and consequently reduced cerebral blood flow. In such cases, the brain may become more vulnerable to fluctuations in perfusion during CPB. Moreover, some chronic condition such as diabetes and atherosclerosis may play an important role as they can influence microcirculation preoperatively [29, 30, 31].

In children with CHD, shunting may also contribute to cerebral hypoperfusion. However, there is a lack of studies in this population investigating cerebral autoregulation in the preoperative period. Moreover, chronic comorbidities are generally rare in young children. Accordingly, the pathophysiology in adults with chronic cardiac conditions likely differs from that in children with CHD. Given the complexity and potential differences in underlying mechanisms, it may not be possible to transfer findings from adult studies to the pediatric population.

### 4.1 Role of Hemodynamic Management

In our study, the duration of impaired autoregulation in relation to the total measurement duration was 14.5% in the delirious vs. 13.9% in the non-delirious group. This represents a significantly shorter period of time compared to the study by Tabone *et al*. [28]. This might be due to the heterogeneity of the patients included in the study by Tabone *et al*. [28] and the longer monitored period. In contrast, the standardized postoperative management at our institution, including close monitoring, establishment of hemodynamic thresholds immediately after postoperative admission, and effective circulatory management, could have contributed to the reduction of the time spent with impaired autoregulation. This is supported by the fact that the periods during which the patients were outside the previously defined hemodynamic thresholds, compared to the total duration of measurements, were very short, and there were no statistically significant differences between both groups. Our rigorous hemodynamic management may have helped maintain stable hemodynamics and, consequently, preserved the integrity of autoregulation parameters. Therefore, although the patients in the delirious group exhibited higher average VIS compared to the non-delirious group, there was no direct influence on the duration of hemodynamic instability.

### 4.2 Incidence of PD

The incidence of PD following congenital cardiac surgery remains two to three times higher compared to the overall incidence of PD in the PICU [5, 6, 7]. This data is consistent with the findings in our study and demonstrates the multifactorial etiology of PD. In this vulnerable group of patients, special attention should be focused on identifying modifiable factors that could influence the onset of delirium.

### 4.3 Other Modifiable Factors

One of these partly modifiable factors is the duration of PICU stay. In our study, it was significantly longer in the delirious group compared to the non-delirious group (6 vs. 4 days, *p* < 0.001), which is in agreement with several other studies [32, 33]. The same trend was observed in the duration of mechanical ventilation which was significantly longer in the delirious group (88 vs. 26 hours, *p* = 0.006). The implementation of early extubation protocols can be beneficial in reducing the length of mechanical ventilation and the stay in the PICU and possibly the incidence of PD [34].

Benzodiazepines and opioids are well-established factors contributing to the development of PD [35, 36]. This corresponds with the data of our study, as the children in delirious group were exposed to higher doses of sedative medications. However, it remains challenging to investigate which of these partly modifiable risk factors plays a predominant role. The children in the delirious group had a more complicated postoperative course with a variety of challenges such as pulmonary hypertension, arrythmias, and respiratory complications, which required prolonged mechanical ventilation, a longer intensive care stay, and deeper levels of sedation. This supports the thesis that postoperative PD has multifactorial and highly complex origins, requiring a more comprehensive approach [13].

### 4.4 Future Perspectives

Another finding in our study, although not statistically significant, was that the rSO2 was higher in the non-delirious group compared to the delirious group—69.6 vs. 65.2%. It would be valuable to explore the duration of rSO2 reduction during the measurement period. In order to accomplish this, an rSO2 threshold could be established to investigate the influence on the development of PD. This suggests that utilizing non-invasive autoregulation monitoring might be beneficial in preventing postoperative neurological complications in certain patient cohorts, for example, in vulnerable patients with comorbidities, complex CHD, multiple surgeries, or prolonged hospital stay. However, further investigations are required to validate this hypothesis. It is important to initiate larger, multicenter studies to more comprehensively understand the role of cerebral autoregulation.

In order to better understand the role of cerebral autoregulation in the development of pediatric delirium, a study with homogeneous subgroups needs to be conducted. To reduce the influence of other established risk factors for PD, it should include only patients with similar durations of mechanical ventilation, sedation doses, and PICU stay.

Excluding the very early postoperative phase may have significantly influenced our findings. Furthermore, it remains unclear whether preoperative factors, such as hemodynamic instability or a hemodynamically significant shunt, had already impaired macro- and microcirculation preoperatively. In such cases, even minimal intraoperative insults could have a significant impact. It is therefore necessary to further investigate the pre- and intraoperative period, as impaired microcirculation in the PICU may play only a secondary role in PD.

This study has several limitations. It was a single-center observational study with a small sample size, which did not allow us to make further subgroup analyses regarding a specific CHD. During the study period, we consistently improved our delirium management in the PICU, which might have influenced the study results. Moreover, we only investigated patients undergoing congenital heart surgery. In our postoperative protocols, we focused on maintaining a mean arterial pressure within strict limits to ensure proper postoperative hemodynamics and actively prevent fluctuations in blood pressure. An additional limitation of this study is the potential inaccuracy of autoregulation thresholds based on NIRS measurements, which may not match the precision of thresholds derived from a complex sampling approach. There might also be other factors that were under-investigated, such as oxygen extraction and metabolic demand, which can influence the interpretation of rSO2 and COx. These factors might have influenced the study findings and must be taken into consideration when assessing the results. We used a threshold for COx of 0.4 based on previous studies. However, this value has not been definitively validated in children with congenital heart disease, and further investigation is needed.

## 5. Conclusion

Postoperative PD continues to have an unclear pathophysiology. It is likely a syndrome with a multifactorial etiology that includes modifiable and non-modifiable factors. Impaired autoregulation might play a role in the development of PD as a modifiable factor in some patient groups. However, further analysis is required to understand the extent of its impact and to determine the most effective strategies for intervention.

## Availability of Data and Materials

The data that support the findings of this study are available from the corresponding author upon reasonable request.
